# *Bartonella henselae* in *Ixodes ricinus* Ticks (Acari: Ixodida) Removed from Humans, Belluno Province, Italy

**DOI:** 10.3201/eid0903.020133

**Published:** 2003-03

**Authors:** Yibayiri O. Sanogo, Zaher Zeaiter, Guiseppe Caruso, Francesco Merola, Stanislav Shpynov, Philippe Brouqui, Didier Raoult

**Affiliations:** * Faculté de Médecine, Marseille, France; † Ospedale San Martino, Belluno, Italy

**Keywords:** *Bartonella henselae* (Houston-1), *Ixodes ricinus*, ticks, Italy, research

## Abstract

The potential role of ticks as vectors of *Bartonella* species has recently been suggested. In this study, we investigated the presence of *Bartonella* species in 271 ticks removed from humans in Belluno Province, Italy. By using primers derived from the 60-kDa heat shock protein gene sequences, *Bartonella* DNA was amplified and sequenced from four *Ixodes ricinus* ticks (1.48%). To confirm this finding, we performed amplification and partial sequencing of the *pap*31 protein and the cell division protein *Fts*Z encoding genes. This process allowed us to definitively identify *B. henselae* (genotype Houston-1) DNA in the four ticks. Detection of *B. henselae* in these ticks might represent a highly sensitive form of xenodiagnosis. *B. henselae* is the first human-infecting *Bartonella* identified from *Ixodes ricinus,* a common European tick and the vector of various tickborne pathogens. The role of ticks in the transmission of bartonellosis should be further investigated.

*Bartonella* species are facultative intracellular bacteria associated with a number of emerging anthropozoonoses. They have been detected in or isolated from diverse vertebrate hosts, including humans (*1*–*3*), various intradomicillary mammals (*4*–*7*), and a wide range of wild animals (*8*,*9*), which serve as natural vertebrate hosts. Various hematophagous arthropods have been implicated in the ecoepidemiology of *Bartonella* species. *B. bacilliformis,* the etiologic agent of Carrión disease, is transmitted by the sand fly (*Lutzomyia verrucarum)* in the Andes Mountains in Peru, Columbia, and Ecuador (*10*). *B. quintana,* the agent of trench fever and bacillary angiomatosis, is found worldwide and is transmitted by the human body louse (*Pediculus humanus)* (*11*).

*B. henselae* is another cosmopolitan emerging human pathogen. This agent was first reported in 1990 in association with bacillary angiomatosis (*12*). The organism was later isolated from the blood of a febrile HIV-positive patient and subsequently described as a new species in 1992 (*1*). *B. henselae* is now recognized as the causative agent of cat-scratch disease (*1*), bacillary angiomatosis, peliosis hepatitis, oculoglandular syndrome, and endocarditis (*13*,*14*). *B.* *henselae* is associated with cats, which serve as its reservoir (*13*,*15*); the cat flea (*Ctenocephalides felis)* was demonstrated to be a vector (*16*). Other *Bartonella*-flea associations are apparent: for example, 61% of rat fleas (*Xenopsylla cheopis*) were found infected with bartonellae, including a known human pathogen, *B. elizabethae* (*7*).

Polymerase chain reaction (PCR) amplification and sequence analysis of various genes are now widely used to differentiate *Bartonella* species. The 16S/23S rRNA intergenic spacer region (*17*), the heat shock protein (*gro*EL) gene (*18*), the citrate synthase gene (*gltA*) (*19*), the riboflavin synthase α-chain gene (*rib*C) (*20*), the cell division protein (*fts*Z) (*21*), and the *pap*31 (*22*) gene sequences were used for detecting, identifying, and classifying the phylogenetic properties and subtyping of *Bartonella* isolates.

Ticks are vectors of more diverse microorganisms than any other arthropod vector (*23*). The sheep tick (*Ixodes ricinus)* is the most common hard tick species in western Europe and has been established as the vector of tick-borne encephalitis virus, *Babesia* sp., *Borrelia burgdorferi*, *Rickettsia helvetica*, and the agent of granulocytic ehrlichiosis, *Anaplasma phagocytophila* (*24*). *I. ricinus* feeds on a large number of vertebrate hosts. The immature stages of *I. ricinus* are found mainly on small-size vertebrates and can readily feed on humans. Ticks have been suspected to transmit *Bartonella* (*25*). However, evidence of *Bartonella* infection in ticks has only recently been reported (*26*,*27*). Although these observations suggest the possibility of *Bartonella* transmission by ticks, more precise identification of these tick-infecting agents is required to establish their zoonotic potential.

## Materials and Methods

### Tick Collection and Identification

During 2000–2001, a total of 271 ticks were removed from asymptomatic persons who visited first aid departments in Belluno Province, Italy, for assistance with tick bites. Ticks were removed with tweezers by grasping their mouthpart and pulling straight out from the skin. The tick-bite site was disinfected, and individual ticks were placed in sterile tubes and kept frozen at –70°C for further study. The ticks were stored on ice during the identification procedure, which was done on the basis of their morphologic features by using standard taxonomic keys.

### Tick DNA Extraction

All ticks were disinfected by immersion into a 70% ethanol solution for 5 min, rinsed with sterile water, and dried in a sterile filter paper. Ticks were then subjected to DNA extraction by using the QiaAmp tissue kit procedure (QIAGEN, GmbH, Hilden, Germany). DNA was extracted from ticks according to the manufacturer’s protocol. To serve as a negative control, DNA of lice from a laboratory colony that had been fed on an uninfected rabbit was extracted, along with tick DNA, to serve as control. DNA was eluted in a final volume of 200 μL and stored at 4°C until studied further.

### PCR Screening of Ticks for the Presence of *Bartonella*

Tick DNA was screened by PCR amplification of the heat-shock protein–encoding gene (*gro*EL) sequences for the presence of *Bartonella*. Primers HSPF1d and BbHS1630.n were used as described (*22*) and are listed in the Table.

**Table:** Primers used for polymerase chain reaction or sequencing

Primer	*Bartonella* species	Primer sequence	Target gene	References	
HSPF1d^a,b^	All	5′-GAACTNGAAGATAAGTTNGAA-3′	groEL	22	
BbHS1630.n^a,b^	All	5′-AATCCATTCCGCCCATTC-3′	groEL	18	
HSP1 ^b^	All	5′-GGAAAAAGTNGGCAATGAAG-3'	groEL	22	
HSP2^b^	All	5′-GCNGCTTCTTCACCNGCATT-3′	groEL	22	
HSPS1^b^	All	5′-AAGCNCCNGGNTTTGGTGA-3′	groEL	22	
HSPS2^b^	All	5′-TCACCAAANCCNGGNGCTT-3′	groEL	22	
HSPF2d^b^	All	5′-GAAAGANCGNGTNGATGAT-3'	groEL	22	
HSPR2d^b^	All	5′-GTNATNAGAAGNCTNGCAAT-3′	groEL	22	
PAPn1^a,b^	*B. henselae* and *B. quintana*	5′-TTCTAGGAGTTGAAACCGAT-3′	pap31	22	
PAPn2^a,b^	*B. henselae* and *B. quintana*	5′-GAAACACCACCAGCAACATA-3′	pap31	22	
PAPns2^a,b^	*B. henselae* and *B. quintana*	5′-GCACCAGACCATTTTTCCTT-3'	pap31	22	
PAPns1^b^	*B. henselae* and *B. quintana*	5′-CAGAGAAGACGCAAAAACCT-3'	pap31	22	
BaftsZF	*B. henselae*	5′-GCTAATCGTATTCGCGAAGAA-3′	ftsZ	This study	
BaftsZR	*B. henselae*	5′-GCTGGTATTTCCAAYTGATCT-3′	ftsZ	This study	
BhftsZ 1393.n	*B. henselae*	5′-GCGAACTACGGCTTACTTGC-3′	ftsZ	21	
Bh ftsZ 1247.p	*B. henselae*	5′-CGGTTGGAGAGCAGTTTCGTC-3′	ftsZ	21	

^a^Amplification primer. ^b^Sequencing primer.

### Subtyping of Detected *Bartonella* with *pap*31 and *fts*Z Partial Sequences

#### Amplification

Primers used for amplification sequencing of each gene are listed in the Table. PCR reactions were performed in a Peltier model PTC-200 thermal cycler (MJ Research, Inc., Watertown, MA). PCR was carried out in a total volume of 50 μL, consisting of 10 pmol of each primer, 0.5 U of ELONGase mix enzyme (GibcoBRL, Cergy Pontoise, France), 20 mM concentration of each deoxynucleoside phosphate, and 1.8 mM of MgCl_2_. Two negative controls were included in the reaction: DNA from uninfected lice, and the master mix with sterile water instead of the DNA template. DNA from a culture of *B. elizabethae* was used as the positive control. The following amplification program was used: a first denaturation step at 94°C for 4 min was followed by 44 cycles of denaturation at 94°C for 30 s, annealing at temperatures corresponding to each gene (53°C for *gro*EL and *pap*31 genes and 55°C for *fts*Z) for 30 s, and a hybridization step at 68°C for 1 min. The amplification reaction was terminated with a further extension step at 68°C for 10 min. PCR products were visualized under UV illumination after electrophoresis migration on a 1% gel agarose stained with ethidium bromide.

#### Sequencing

PCR products were purified by the QIAquick PCR purification kits (QIAGEN, GmbH) as recommended by the manufacturer. Primers used for the sequencing of each gene are listed in the Table. PCR products were sequenced in both directions by using the d-Rhodamine Terminator Cycle Sequencing Ready Reaction kit (PerkinElmer, Inc., Coignières, France) according to the manufacturer’s recommendations. Sequencing products were resolved in an Applied Biosystem automatic sequencer model 3100 (PerkinElmer).

#### Sequence Analysis

Nucleotide sequences were edited with the Autoassembler (version 1.4; Perkin Elmer) package. Multiple alignment with other *Bartonella* sp. sequences available from GenBank was carried out by using the Clustal W program (*28*).

## Results

Of the 271 ticks collected from patients, 268 were *I. ricinus* (98.9%); the other specimens were one female *I. hexagonus* (0.4%), one female *Rhipicephalus sanguineus* (0.4%), and one female *I. ventalloi* (0.4%). Most of the ticks were nymphs (142; 52.3%), followed by females (115; 42.4%); larva (10; 3.6%), and males (1; 0.4%).

### PCR Screening of Ticks for *Bartonella*

By using primers HSPF1d and BbHS1630.n, a single band of PCR product of approximately 1,490 bp was amplified and sequenced in four *I. ricinus* ticks (two females and two nymphs) (1.48%). No amplification product was yielded from the negative controls. We used the BLAST tool (available from: URL: http://www.ncbi.nlm.nig.gov/BLAST/); the search of the 1,422-base sequenced fragment from all four ticks revealed a 100% homology with *B. henselae* Houston-1 (GenBank accession no. AF014829).

### Subtyping of *Bartonella henselae*

Amplification of the *pap*31 and *fts*Z partial sequences yielded 257-bp and 885-bp, fragments, respectively. Sequences of these products had 100% identity with those of *B. henselae* Houston-1 (GenBank accession nos. AF001274 and AF061746, respectively).

## Discussion

Recently, vector biologists and epidemiologists have suggested that ticks may have a role in *Bartonella* transmission (*29*). In 1996 Kruszewska et al. reported the preliminary finding of a *Bartonella* strain in *I. ricinus* ticks from a park in Walz, Poland (*26*). Unfortunately, the strain has not been further characterized. In a study conducted in the Netherlands, the 16S rRNA gene sequences of an unspecified *Bartonella* were amplified in >70% of *I. ricinus* ticks removed from roe deer (*27*). Such a high prevalence of *Bartonella* in ticks is surprising and may be because ticks were collected while they were feeding on bacteriemic hosts (*30*,*31*). According to Schouls et al., none of the *Bartonella* organisms detected was a known human pathogen (*27*). More recently, different *Bartonella* sp., including *B. quintana, B. henselae, Bartonella* strain cattle-1, *B. washoensis,* and *B. vinsonii* subsp. *Berkhoffii*, have been detected in 19.2% of *I. pacificus* ticks collected in California by amplification and sequencing of a fragment of the *glt*A gene (*32*).

In this study, we report the detection of *B. henselae* in four *I. ricinus* ticks (1.4%) removed from persons in Italy. Because the primers used in the screening PCR generate rather large PCR fragments (1,490 bp), this prevalence could be expected to be greater (usually, the longer the PCR product, the lower the sensitivity). DNA from positive samples was further characterized by using the *gro*EL, the *pap*31, and the *Fts*Z genes to establish their relationship with known *Bartonella* sp. and subsp. On the basis of the 16S rRNA genes and immunogenic characteristics, Drancourt et al. (*33*) suggested the presence of two variants of *B. henselae.* Ribosomal genes such as the 16S rRNA genes are, however, highly conserved within bacteria and can pose the risk of unspecific amplification. Protein-coding genes exhibit a higher degree of sequence variation and thus can be targeted as tools for differentiating strains of the same species. Although the two genogroups of *B. henselae,* Marseille and Houston-1, are closely related, and the respective pathogenicity spectrum of the two serotypes has not been established, the serotypes could be differentiated on the basis of sequences of the *gro*EL, C-terminal region of the *ftsz* gene and the *pap*31 gene (*21*,*22*).

In northwestern Italy, about 89% of the ticks found to parasitize people were *I. ricinus* (*34*). In our study, four different species of ticks were recorded from humans, and *I. ricinus* was recorded most frequently (98.9%). All the active life stages of *I. ricinus* were represented.

Experimental studies and epidemiologic observations have suggested that ticks may play a role in the transmission of *Bartonella* sp. *Dermacentor andersoni* was proven to be a competent vector of *B. bacilliformis* in the experimental infection of nonhuman primates many years ago (*35*). *B. vinsonii* subsp. *berkhoffii* infection was correlated with heavy tick infestation of dogs (*36*). The cat flea (*Ctenocephalides felis)* is the main arthropod vector of *B. henselae* with cats serving as the main vertebrate reservoirs. Although finding *B. henselae* in ticks might suggest another possible reservoir, *I. ricinus*–like ticks have a very broad host range and are known to infest cats. In our study, none of the persons from whom the positive ticks were collected exhibited symptoms associated with *B. henselae* infection. However, serum specimens from these patients have not been tested. Because bacteremia levels in cat-scratch disease patients have never been consistently demonstrated (*37*), the tick was unlikely to have acquired *B. henselae* from feeding on patients with asymptomatic cat-scratch disease. Nevertheless, a number of wild animals, including the preferred hosts of both adults and immature stages of *I. ricinus* (*38*) have been found to be infected with *Bartonella* sp (*8*,*39*). Questing ticks have been found infected with *Bartonella,* including *B. henselae* (*32*). Furthermore, ticks were suspected of being vectors of *B. henselae* in an epidemiologic study conducted by Lucey et al. in 1992 (*25*). These authors reported *B. henselae* bacteremia levels in patients who recalled a tick bite but had no history of contact with cats. Ticks were also reported as possible source of infection in some human cases of concurrent infection of the central nervous system by *Borrelia burgdorferi* and *Bartonella henselae* (*40*). The evidence that ticks may serve as *Bartonella* vectors appears to be rapidly accumulating.

In conclusion, we have confirmed that ticks feeding on humans were infected with the agent of cat-scratch disease, *B. henselae* (Houston-1). The source of infection of the ticks was not determined. No case of transmission to humans was observed. However, our findings suggest that the ticks were naturally infected. These results support the argument that ticks are involved in the transmission of *Bartonella* organisms and represent a potential source of infection for persons exposed to tick bites. Therefore, we encourage further investigation of ticks as vectors of human pathogenic *Bartonella* strains.
